# Changes in the Brain Accumulation of Glucocorticoids in abcb1a-Deficient CF-1 Mice

**DOI:** 10.1111/j.1365-2826.2012.02353.x

**Published:** 2012-11

**Authors:** B L Mason, C M Pariante, S A Thomas

**Affiliations:** * Institute of Pharmaceutical Science, King’s College LondonLondon, UK; † Section and Laboratory of Stress, Psychiatry and Immunology (SPI-Lab), Institute of Psychiatry, King’s College LondonLondon, UK

**Keywords:** blood–brain barrier, choroid plexus, glucocorticoids, P-glycoprotein, hypothalamic-pituitary-adrenal axis

## Abstract

The multidrug resistance transporter, P-glycoprotein (P-gp), contributes to highly lipophilic molecules penetrating the brain from the blood at a much lower rate than expected, and has numerous substrates, inhibitors and modulators. The drug-transporting isoform of P-gp is coded by a single human gene, ABCB1, and shares 80% homology with the murine drug-transporting isoforms, abcb1a and abcb1b, which share 92% homology with each other. Although these murine isoforms are highly similar, there are known affinity differences between the isoforms, and the localisation of the two isoforms in the brain is also disputed. Studies using mice genetically modified to be deficient in one or both isoforms of P-gp have also resulted in conflicting data. The contribution of the abcb1a isoform, which is considered to contribute most to the central nervous system (CNS)-protective role of P-gp, is investigated in the present study using CF-1-abcb1a(−/−) mice and the well-established brain/choroid plexus perfusion technique. Twenty-minute *in situ* brain/choroid plexus perfusions in CF-1-abcb1a(−/−) mice indicated the increased accumulation of [^3^H]cortisol, [^3^H]corticosterone and [^3^H]dexamethasone in most of the brain regions examined compared to CF-1-abcb1a(+/+) mice. Taken together with our earlier published studies in abcb1a/b(−/−) mice, these data strongly suggest that the *in vivo* CNS accumulation of glucocorticoids obtained using single knockout strains [e.g. abcb1a(−/−)] cannot be directly compared with those obtained in double knockout strains [e.g. abcb1a/b(−/−)].

The multidrug resistance transporter P-glycoprotein (P-gp) has been determined to be a major contributor to the phenomenon of highly lipophilic molecules penetrating the brain from the blood at a much lower rate than expected (1). P-gp is expressed in a number of tissues, including the liver, adrenal glands and, importantly, in human and murine cerebral capillary endothelial cells, where it is found to actively transport substrates across the luminal (apical) membrane of the endothelial cell into the blood (2–5). In humans, the drug-transporting isoform of P-gp is coded by the gene ABCB1 (5–7) and, in rodents, abcb1a and abcb1b code for the drug-transporting isoform, which together share 80% homology with the human form ABCB1 and 92% homology with each other (6,8). Although these isoforms are highly similar, there are known affinity differences between the isoforms; for example, abcb1b was shown to confer high drug resistance to the P-gp substrate, colchicine, *in vitro*, whereas abcb1a conferred very low drug resistance. Conversely, abcb1a more effectively conferred a high resistance to another P-gp substrate, vinblastine, compared to abcb1b (8).

Importantly, the localisation of the two rodent isoforms in the brain is also disputed. P-gp was detected in isolated human and rat brain capillaries (9), and both abcb1a and abcb1b have been found in murine brain, with more abcb1a detected than the abcb1b (4). The abcb1a isoform was later localised to the capillary endothelial cells (6,10–12). Work performed *in vitro* determined that abcb1b was expressed in the rat brain parenchyma, rather than the endothelial cells (13), and cultured rat brain endothelial cells have shown an expression of abcb1b *in vitro* when none was detected *in vivo* (14). More recent work has failed to find abcb1b in isolated rat brain capillaries, although the expression of abcb1b was found in its control brain samples, including hippocampal tissue, leading to the conclusion that abcb1b expression could be specific to certain brain regions (12). Therefore, it is possible that abcb1b may be present in specific sites within the brain, although it is not present at the level of the blood–brain barrier. Interestingly, there is minimal abcb1b in the mouse brain hippocampus (15), although abcb1a can be found in the dentate gyrus (16); thus, there appear to be differences in the regional expression of the different isoforms. P-gp has also been detected in rodent choroid plexus (17,19), likely localised to the apical or subapical membrane. The expression of P-gp at the choroid plexus, a fundamental component of the blood-cerebrospinal fluid (CSF) barrier (18,21), may also significantly affect the accumulation of substrates in different regions of the central nervous system (CNS).

Of particular interest to our research group are the conclusions reached when the transport of the glucocorticoid hormones cortisol, dexamethasone and corticosterone by P-gp has been studied (21–32). For example, dexamethasone was shown to be transported *in vitro* by abcb1b and showed little to no transport by abcb1a (26); however, *in vivo* tissue accumulation of dexamethasone was increased in abcb1a-deficient mice compared to abcb1a-deficient mice (27). It is important to note that the differing tissue expression of these isoforms will mean that the *in vivo* tissue accumulation of glucocorticoids can be significantly affected by the method used in the study. After systemic administration, in which radiolabelled glucocorticoids were administered s.c. to adrenalectomised mice and allowed to distribute for 1 h, abcb1a(−/−) mice were shown to have a higher brain-to-blood ratio of [^3^H]dexamethasone and [^3^H]cortisol compared to abcb1a(+/+) mice, with no differences in the brain accumulation of [^3^H]corticosterone between strains, thus implicating P-gp in the efflux of [^3^H]dexamethasone and [^3^H]cortisol from the murine brain (21,27). Because these mice were adrenalectomised, the only glucocorticoids present would be those administered radiolabelled compounds and the blood/plasma radioactivity concentrations did not differ between strains, suggesting that the metabolism of the glucocorticoids was not significantly affected by the removal of the abcb1a isoform. This finding was supported by autoradiography showing the presence of radioactive binding of [^3^H]dexamethasone to glucocorticoid receptors in the brains of abcb1a(−/−) but not abcb1a(+/+) mice (27), and strong binding in the hippocampus (likely driven by mineralocorticoid receptor expression) and mild increases in the rest of the brain tissue of [^3^H]cortisol in abcb1a(−/−) mice but not in abcb1a(+/+) mice, as well as *in vitro* transport of cortisol by human ABCB1A in LLC-PK1 cell lines (21). However, higher concentrations of both [^3^H]cortisol and [^3^H]corticosterone were detected in the brains of adrenally-intact abcb1a/b(−/−) mice compared to abcb1a/b(+/+) mice (28), now implicating P-gp in the efflux of [^3^H]corticosterone, as well as [^3^H]cortisol, from the murine brain after total ablation of P-gp from the body. *In vivo* systemic administration of radiolabelled glucocorticoid to adrenally-intact mice and the subsequent assessment of their distribution to the CNS will be complicated by the disposition of the substrate into peripheral tissues, especially considering the lipophilic nature of these glucocorticoids, their breakdown after repeated circulation through the body, and the subsequent unknown radioactive blood residues, thus making the data somewhat difficult to interpret. Additionally, abcb1a/b(−/−) mice have been shown to have attenuated stress responses (lower levels of plasma adrenocorticotrophic hormone at basal and stress conditions and lower plasma corticosterone levels at the diurnal nadir but not at basal conditions) compared to abcb1a/b(+/+) mice (30), and abcb1a/b(−/−) mice have also been noted to have lower plasma corticosterone concentrations compared to abcb1a/b(+/+) mice (30). This would suggest that complete ablation of P-gp has significant effects on the regulation of the HPA axis *in vivo*, and, because corticosterone is the endogenous glucocorticoid for rodents, this would also implicate abcb1b in the access of corticosterone to the brain, in accordance with the findings of Uhr *et al.* (28). Our previous work using the well-established brain/choroid plexus perfusion method in which radiolabelled glucocorticoids are administered over a 20-min period to adrenally-intact mice has shown that dexamethasone accumulation is increased in the frontal cortex, hippocampus and hypothalamus of abcb1a/b(−/−) mice, and cortisol accumulation is increased in the hypothalamus of abcb1a/b(−/−) mice compared to abcb1a/b(+/+) mice, with corticosterone accumulation not being affected by the absence of P-gp, with no changes seen in the accumulation of any of these glucocorticoids in the choroid plexus (31,32).

As a result of these conflicting findings regarding the contribution of the two drug-transporting isoforms of P-gp, and our previous findings using abcb1a/b(−/−) mice showing the increased accumulation of glucocorticoids in a limited number of brain regions, we investigated the contribution of the abcb1a isoform only, which is considered to contribute to the CNS-protective role of P-gp. We used CF-1 abcb1a(−/−) mice and the well-established brain/choroid plexus perfusion technique in anaesthetised mice that allows for the perfusion of both cerebral hemispheres and the consequent examination of molecule movement across both the blood–brain and blood–CSF barriers (31). The benefits of this method include the elimination of first-pass metabolism of substrates, thereby ensuring that intact substrate is administered to the CNS; control of the perfusion media (including the removal of additional plasma binding factors that may change the overall concentration of free glucocorticoid present in the bloodstream); and the co-administration of a vascular-space marker indicating that the blood–CNS barriers are indeed intact and functioning, as well as quantifying the vascular volume that will be a proportion of all brain samples investigated. Additionally, our perfusion rate of 5.0 ml/min has been previously validated to ensure full perfusion of the vasculature without disruption to the barriers (33).

## Materials and methods

### Animals

Adult male CF-1® abcb1a(+/+) mice and Crl:CF1- abcb1a(−/−) mice were obtained from Charles River Laboratories (Margate, UK). All animals were maintained under standard conditions of temperature and lighting and given food and water *ad lib*.

### Materials

[^3^H]cortisol (74.0 Ci/mmol), [^3^H]corticosterone (79.0 Ci/mmol) and [^3^H]dexamethasone (89.0 Ci/mmol) were purchased from GE Healthcare (formerly Amersham Biosciences; Little Chalfont, UK). [^14^C]Sucrose (0.49 Ci/mmol) was purchased from Moravek Biochemicals (Brea, CA, USA). All other materials were purchased from Sigma (St Louis, MO, USA), unless stated.

### Procedures

All procedures were performed within the guidelines of the Animals (Scientific Procedures) Act, 1986.

#### *In situ* brain/choroid plexus perfusion technique

Adult male mice (25–32 weeks and 25 g–35 g) were anaesthetised with a medetomidine hydrochloride (2 mg/kg, i.p.) and ketamine hydrochloride solution (150 mg/kg, i.p.) and heparinised (100 U, i.p.). The body cavity was opened and the left ventricle cannulated with a fine needle (25-gauge) connected to a perfusion circuit. A Watson-Marlow peristaltic pump (323S/RL; Watson-Marlow, Cornwall, UK) was used to perfuse the heart *in situ* with a modified Krebs-Henseleit mammalian Ringer solution (33), which was warmed (37 °C) and oxygenated (95% O_2_; 5% CO_2_). With the start of perfusion (5.0 ml/min), the right atrium was sectioned to create an open circuit and allow drainage of the artificial plasma. A 2.5-min pre-drug perfusion of artificial plasma ensured removal of endogenous glucocorticoids from the brain vasculature. [^3^H]Cortisol (3.6 nm), [^3^H]corticosterone (3.8 nm) or [^3^H]dexamethasone (3.9 nm), along with [^14^C]sucrose (vascular space marker; 0.5–1.0 nm), was then administered by a slow-drive syringe pump (model 22; Harvard Apparatus, Edenbridge, UK) into the artificial plasma. After the desired isotope perfusion period (20 min), the mouse was decapitated and the brain was removed and specific samples [frontal cortex, hypothalamus, hippocampus, cerebellum, choroid plexus (IV ventricle) and pituitary gland, comprising anterior and posterior lobes] were dissected and weighed. All brain samples, together with 100-μl artificial plasma samples, were prepared for liquid scintillation counting as described below.

#### Capillary depletion step

To assess how much of drug has actually entered the brain tissue, rather than accumulated within the cerebral capillary endothelial cells, the capillary depletion method was performed (34). The brain remaining after dissection of the individual samples was weighed and homogenised with five strokes in the presence of a capillary depletion buffer [10 mm HEPES, 140 mm NaCl, 4 mm KCl, 2.8 mm CaCl_2_(6H_2_O), 1 mm MgSO_4_(7H_2_O), 1 mm NaH_2_PO_4_(2H_2_0), 10 mm g/l glucose (volume × three brain weight composition)]. A 26% dextran solution [MW 60 000–90 000 (volume × four brain weight)] was then added to the homogeniser and combined with five strokes. Two 100-μl aliquots of this whole brain homogenate were taken and weighed, and the rest of the homogenate was centrifuged at 5400 ***g*** for 15 min at 4 °C. The endothelial cell-enriched pellet and the supernatant containing brain parenchyma and interstitial fluid were then separated and weighed. Homogenate, supernatant and pellet samples were prepared for radioactive analysis as described below.

#### Liquid scintillation counting

All samples were solubilised over approximately 48 h in 0.5 ml of Solvable (PerkinElmer Life and Analytical Sciences, Boston, MA, USA). All samples were vortexed, 3 ml of scintillation counting fluid (Lumasafe; PerkinElmer Life and Analytical Sciences) was then added and the samples were vortexed again. The samples were then placed in a Packard TriCarb 2100 or 2900TR (PerkinElmer; Beaconsfield, UK) liquid scintillation counter for estimation of [^3^H] and [^14^C] radioactivities. All results were corrected for background radioactivity.

#### Expression of results

The concentration of [^3^H] or [^14^C] radioactivity in the brain tissue, homogenate, supernatant and pellet (d.p.m./g) is expressed as a percentage of that in the artificial plasma (d.p.m./ml) and is referred to as R_Tissue_%. R_Tissue_% values of the radioactive glucocorticoids were corrected with the R_Tissue_ values of [^14^C]sucrose and termed R_CorrTissue_% or ‘vascular space-’ or ‘extracellular space-corrected’. Correcting the distribution in this way allowed simple comparisons to be made between the different strains, as well as between the different radiolabelled glucocorticoids. However, these corrections could only be used when the [^14^C]sucrose values did not differ significantly between groups.

### Statistical analysis

Student’s t-tests were used to compare the [^14^C]sucrose values and the uptake of [^3^H]glucocorticoids between strains. In all cases, P < 0.05 was considered statistically significant.

## Results

The [^14^C]sucrose values were not significantly different between strains for any sample (P > 0.05; Student’s t-test); therefore, the vascular space-, extracellular space- and sucrose-corrected [^3^H]glucocorticoid values are presented.

### Brain regions

**Fig 1:**
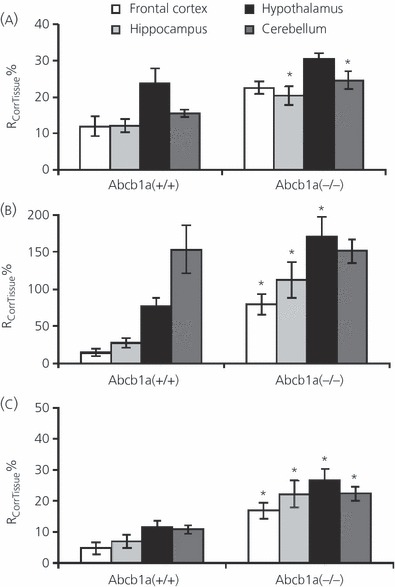
R_CorrTissue_% (ml/100g) of [^3^H]cortisol (a), [^3^H]corticosterone (b) or [^3^H]dexamethasone (c) in the brain regions after a 20-min perfusion in abcb1a(+/+) and abcb1a(−/−) mice (n = 5/strain); *Statistically significant (P < 0.05) compared to abcb1a/b(+/+) values.

The uptake of [^3^H]cortisol was significantly higher in the hippocampus and cerebellum (P = 0.0447, P = 0.024, respectively; Student’s t-test, vascular space-corrected; Fig. 1A) of abcb1a(−/−) mice compared to abcb1a(+/+) mice. The uptake of [^3^H]cortisol tended to be higher in the frontal cortex of abcb1a(−/−) mice compared to abcb1a(+/+) mice; however, this failed to meet statistical significance (P = 0.052; Student’s t-test, vascular space-corrected). The uptake of [^3^H]corticosterone was significantly higher in the frontal cortex, hippocampus and hypothalamus (P = 0.002, P = 0.009, P = 0.011, vascular space-corrected; Fig. 1B) of abcb1a(−/−) mice compared to abcb1a(+/+) mice, and the uptake of [^3^H]dexamethasone was significantly higher in the frontal cortex, hippocampus, hypothalamus and cerebellum (P = 0.006, P = 0.014, P = 0.006, P = 0.003, respectively; Student’s t-test, vascular space-corrected; Fig. 1C) of abcb1a(−/−) mice compared to abcb1a(+/+) mice.

### Choroid plexus and pituitary gland

**Fig 2:**
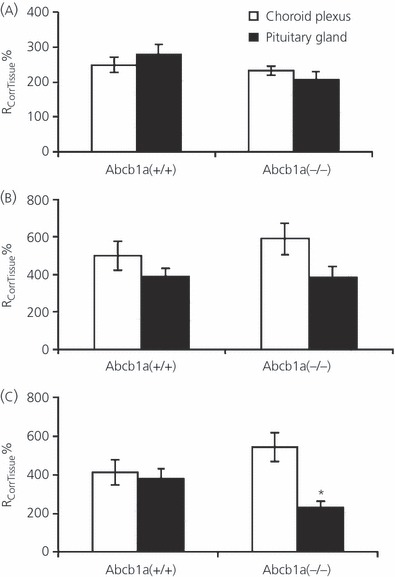
R_CorrTissue_% (ml/100g) of [^3^H]cortisol (a), [^3^H]corticosterone (b) or [^3^H]dexamethasone (c) in the choroid plexus and pituitary gland after a 20-min perfusion in abcb1a(+/+) and abcb1a(−/−) mice (n = 5/strain); *Statistically significant (P < 0.05) compared to abcb1a/b(+/+) values.

The uptake of [^3^H]dexamethasone was significantly lower in the pituitary gland of abcb1a(−/−) mice compared to abcb1a(+/+) mice (P = 0.041; Student’s t-test, extracellular space-corrected; Fig. 2C); in contrast, the uptake of [^3^H]cortisol or [^3^H]corticosterone was not significantly different in the pituitary gland between strains (Fig. 2A,B, respectively). The uptake of [^3^H]cortisol, [^3^H]corticosterone or [^3^H]dexamethasone was not significantly different in the choroid plexus of abcb1a(+/+) mice compared to abcb1a(−/−) mice.

### Capillary depletion analysis

**Fig 3:**
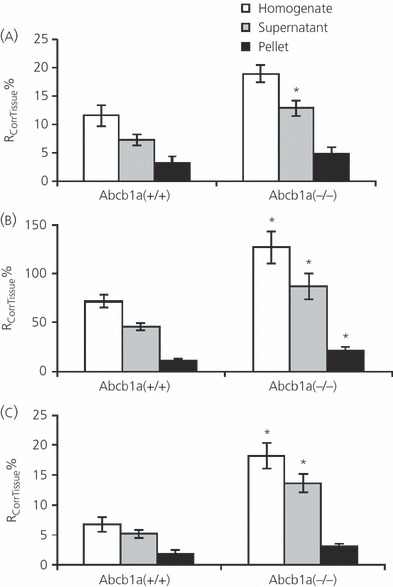
R_CorrTissue_% (ml/100g) of [^3^H]cortisol (a), [^3^H]corticosterone (b) or [^3^H]dexamethasone (c) in the products of capillary depletion analysis after a 20-min perfusion in abcb1a(+/+) and abcb1a(−/−) mice (n = 5/strain); *Statistically significant (P < 0.05) compared to abcb1a/b(+/+) values. The whole brain homogenate is separated into a brain parenchyma containing supernatant and a capillary endothelial cell-enriched pellet.

Capillary depletion analysis revealed a significantly higher accumulation of [^3^H]cortisol in the brain parenchyma-containing supernatant fraction (P = 0.043; Student’s t-test, sucrose-corrected; Fig. 3A) of abcb1a(−/−) mice compared to abcb1a(+/+) mice. A significantly higher accumulation of [^3^H]corticosterone was detected in the whole brain homogenate, the brain parenchyma-containing supernatant fraction and the capillary-enriched pellet (P = 0.015, P = 0.016, P = 0.007, respectively; Student’s t-test, sucrose-corrected; Fig. 3B) and a significantly higher accumulation of [^3^H]dexamethasone was also detected in the homogenate, supernatant and pellet (P = 0.001, P < 0.001, P = 0.017, respectively; Student’s t-test, sucrose-corrected; Fig. 3C) of abcb1a(−/−) mice compared to abcb1a(+/+) mice.

## Discussion

Twenty-minute *in situ* brain/choroid plexus perfusions in CF-1-abcb1a(−/−) mice indicated an increased accumulation of [^3^H]cortisol in the hippocampus and cerebellum; an increased accumulation of [^3^H]corticosterone in the frontal cortex, hippocampus and hypothalamus; and an increased accumulation of [^3^H]dexamethasone in the frontal cortex, hippocampus, hypothalamus and cerebellum compared to CF-1-abcb1a(+/+) mice. The accumulation of [^3^H]cortisol, [^3^H]corticosterone and [^3^H]dexamethasone was not significantly different in the choroid plexus of CF-1-abcb1a(−/−) mice compared to CF-1-abcb1(+/+) mice, and the accumulation of [^3^H]dexamethasone was significantly reduced in the pituitary gland of CF-1-abcb1a(−/−) mice compared to CF-1-abcb1a(+/+) mice, although accumulation in the pituitary gland was not significantly different with regard to [^3^H]cortisol or [^3^H]corticosterone. Capillary depletion analysis, a method in which a capillary-enriched pellet can be separated from the brain parenchyma and interstitial fluid (supernatant fraction) and therefore analysed separately, supported the evidence indicating that the absence of the abcb1a isoform of P-gp at the blood–brain barrier does lead to an increased accumulation of these [^3^H]glucocorticoids in the brain. There was significantly more [^3^H]cortisol, [^3^H]corticosterone and [^3^H]dexamethasone in the brain parenchyma (supernatant fractions) of CF-1-abcb1a(−/−) mice compared to CF-1-abcb1a(+/+) mice, although the only glucocorticoid that had a significantly greater distribution in the capillary endothelial cell-enriched pellet of CF-1-abcb1a(−/−) mice compared to CF-1-abcb1a(+/+) mice was [^3^H]corticosterone.

These data are consistent with previously published work in FVB-abcb1a-deficient mice from other laboratories that found significantly more [^3^H]cortisol and [^3^H]dexamethasone in the brains of these single isoform-deficient mice (21). Because the abcb1a-deficient mice used in the present study are derived in CF-1 mice, compared to those abcb1a/b-deficient mice used by Karssen derived in FVB mice, it is encouraging that the data are consistent regarding the role of abcb1a in the CNS distribution of [^3^H]cortisol. This further supports the hypothesis that cortisol is a substrate for P-gp, which is supported by *in vitro* work that previously indicated an interaction between P-gp and cortisol in human and hamster cells (21–24), the previously mentioned *in vivo* data by Karssen *et al.* (21), as well as our own work showing an increased accumulation of [^3^H]cortisol in the hypothalamus of abcb1a/b(−/−) mice compared to abcb1a/b(+/+) mice after *in situ* brain/choroid plexus perfusion (32). Additionally, the increased brain penetration of [^3^H]dexamethasone in abcb1a-deficient mice in the present study is also consistent with previously published work (27). By contrast to the previously published studies (21,31), these new data now suggest that corticosterone may be a substrate for the abcb1a isoform of P-gp. Our previous study found no significant differences in the brain accumulation of [^3^H]corticosterone between abcb1a/b-deficient mice and wild-type controls (32). Potentially, these differences could also have been related to strain differences because Karssen *et al.* (21) did use the FVB-abcb1a(−/−) mice, whereas our mice are derived from the CF-1 strain.

The abcb1a-deficient mice provide an opportunity to attempt to elucidate the potential contribution of the abcb1a isoform of P-gp because it is the isoform believed to be most densely expressed at the blood–brain barrier (6,10–12,14). Importantly, up-regulation of the abcb1b isoform has been seen in the liver and kidneys of abcb1a(−/−) mice compared to abcb1a(+/+) mice (35), and thus potentially confounds the evidence obtained using these single knockout mice depending on the method used to evaluate blood–brain barrier penetration. However, the possible increased activity of abcb1b in abcb1a(−/−) mice may help clarify the true role of abcb1b in the CNS accumulation of these [^3^H]glucocorticoids. If there is no difference in the brain accumulation of these [^3^H]glucocorticoids in a particular brain region in the CF-1-abcb1a(−/−) and CF-1-abcb1a(+/+) mice, this would further suggest that abcb1b also does not play a role in the accumulation of the substrate being tested. Importantly, differences in the transport and inhibition properties between the two isoforms of murine P-gp have previously been identified, which also varies dependent on the substrate under study (36). These differences could be a result of the two distinct binding sites of P-gp because Garrigues *et al.* (37) has characterised two distinct (but overlapping) pharmacophores with the P-gp substrate vinblastine binding to Pharmacophore 2, and the P-gp inhibitor, verapamil, binding to Pharmacophore 1 (38). Thus, although these isoforms share 92% homology (8), their substrate and inhibitor affinities may differ, which is further complicated by differing tissue expression. Dexamethasone has been shown to be a substrate for abcb1b *in vitro*; however, the same study also found that cell lines expressing abcb1a were not dexamethasone-resistant (26). This contradicts the *in vivo* data in which the accumulation of [^3^H]dexamethasone was increased in abcb1a(−/−) mice compared to abcb1a(+/+) mice (27) but possibly highlights a true affinity of [^3^H]dexamethasone for abcb1b. Interestingly, there were minimal differences in [^3^H]dexamethasone CNS accumulation when comparing CF-1-abcb1a(+/+) mice with FVB-abcb1a/b(+/+) mice, suggesting that [^3^H]dexamethasone has a highly similar CNS penetration in these two strains, further supporting the hypothesis that dexamethasone has a strong affinity for the abcb1a isoform. The data obtained in the present study now indicate that cortisol may have a higher affinity for the abcb1a isoform of P-gp than for the abcb1b isoform as a result of the greater number of brain regions affected by the absence of abcb1a compared to the number affected in the absence of abcb1a/b. We are aware that the *in situ* model used in the present study cannot fully clarify the contribution of either abcb1a or abcb1b in the transport of glucocorticoids. Complementary studies in *in vitro* systems using transfected murine abcb1 and abcb1b could provide helpful data to elucidate the role these isoforms play with regards to glucocorticoid transport. We do feel, however, that the *in situ* model provides a system that more closely mimics the physiological movement of glucocorticoids across the blood–CNS barriers and therefore provides valuable evidence for the role that abcb1 may play in regulating glucocorticoid accumulation in the CNS.

Rather than indicating a stronger affinity for abcb1a over abcb1b, these data may also support the hypothesis that the abcb1a isoform is the isoform that is most densely expressed at the BBB. This therefore emphasises that the complete ablation of P-gp, such as in the FVB-abcb1a/b(−/−) mice, leads to disruption of the brain accumulation of P-gp substrates by other mechanisms, such as the changed liver clearance by P-gp or the hampered excretion of corticosterone from the adrenal gland, as proposed previously (32). Importantly, these inconsistencies may also suggest the up-regulation of additional transporters in the mice wholly deficient for P-gp compared to single isoform-deficient mice. We have also recently found an interaction between cortisol and corticosterone and some unidentified transporters in FVB-wild-type mice, although many of these interactions were seen in the presence of supraphysiological concentrations of cortisol and corticosterone (31). The efflux transporter, breast cancer resistance protein (BCRP), is known to be up-regulated in CF-1-abcb1a(−/−) mice and corticosterone has been shown to inhibit the transport of BCRP substrates, although it is not considered to be a substrate itself (39). However, interestingly, the addition of the P-gp substrate, colchicine, or the P-gp and BCRP substrate, daunorubicin, caused an increase in the uptake of [^3^H]corticosterone in the brains of rats (2), suggesting that there is an interaction between corticosterone and P-gp or BCRP.

The differences between our previously published work in FVB-abcb1a/b(−/−) and FVB-abcb1a/b(+/+) mice and the present data obtained in CF-1-abcb1a(−/−) and CF-1-abcb1a(+/+) mice, specifically the additional brain regions with increased uptake of [^3^H]cortisol in CF-1-abcb1a(−/−) mice and the increased uptake of [^3^H]corticosterone in CF-1-abcb1a(−/−) mice compared to abcb1a(+/+) mice, could very likely highlight strain differences in the uptake of [^3^H]glucocorticoids normally occurring between these mice. Differences between strains in the expression of transporters have been noted. For example, MRP2 was found to be absent from the cerebral capillary endothelial cells of FVB mice, whereas MRP2 expression was detected in the capillaries of C57Bl/6J, Swiss and SVJ mice (40); therefore, FVB mice would already possess an increased accumulation of MRP2-exclusive substrates compared to other mouse strains. This increased accumulation (as a result of decreased efflux) could also affect other unidentified transporters or those compounds that share affinity with MRP2 and additional transporters; thus, this differing baseline must be kept in mind when comparing data obtained in FVB mice with data obtained in other strains. Interestingly, the accumulation of [^3^H]cortisol in the hypothalamus of CF-1 wild-type mice was substantially greater than the [^3^H]cortisol accumulation previously obtained in the FVB wild-type mice (32), perhaps highlighting the existence of another efflux transporter present in the FVB mice that is highly expressed in the hypothalamus with cortisol as a substrate. As noted previously, it has been hypothesised that the abcb1b isoform of P-gp may be expressed in a brain region-specific manner (12), which would be consistent with our present and past data (31,32).

Interestingly, the uptake of [^3^H]dexamethasone was significantly reduced in the pituitary gland of CF-1-abcb1a(−/−) mice compared to CF-1-abcb1a(+/+) mice, which is in contrast to the results previously found with FVB-abcb1a/b(−/−) mice, where there was no significant difference in the uptake of [^3^H]dexamethasone compared to FVB-abcb1a/b(+/+) mice. This could indicate a functional difference for P-gp in the pituitary gland, with it possibly being expressed on the opposing cellular membrane, or it could possibly indicate the up-regulation of mdr1b, or a compensatory unidentified efflux transporter, in this region. Changes in the expression of abcb1b have previously been seen in abcb1a(−/−) mice, although these were localised to certain tissues (liver, kidney) by Schinkel *et al.* (35) and changes in the expression of abcb1b in whole brain of abcb1a(−/−) were not seen; however, this does not rule out possible changes in expression in particular brain regions, which was not analysed at the time. Because we know that [^3^H]dexamethasone accumulation in the CNS is highly regulated by P-gp, this may indicate that abcb1b is indeed expressed in this tissue and that [^3^H]dexamethasone does have an affinity for the abcb1b isoform of P-gp, as previously seen in an *in vitro* system (26).

These data, in conjunction with our previously published data (31,32), strongly suggest that the *in vivo* CNS accumulation of glucocorticoids is regulated by P-gp in a complicated manner. We cannot determine from the current results which potential factor (i.e. true substrate affinity, tissue distribution, strain variance, compensatory mechanisms) is the determining difference; however, it does highlight that the results obtained with respect to the transport of glucocorticoids by P-gp must be reviewed in the context of how the data were collected.
